# The role of mechanosensory T cells for stimulus encoding in the local bend network of the leech

**DOI:** 10.1186/1471-2202-16-S1-P132

**Published:** 2015-12-18

**Authors:** Friederice Pirschel, Oliver Kuehn, Jutta Kretzberg

**Affiliations:** 1 Computational Neuroscience, University of Oldenburg, D-26111 Oldenburg, Germany

## 

Accurate behavioral responses to sensory stimuli require reliable encoding and processing of stimulus properties in the underlying neuronal network. The relatively simple nervous system of the leech is able to react with surprising precision when the skin is touched: With their local bend response [1], leeches discriminate behaviorally between touch location differences of 9° [2], corresponding to a distance of less than 1 mm. The underlying network consists of one layer of sensory neurons, approximately 20 interneurons and a layer of motor neurons [1]. Studies investigating the local bend mainly focused on one of the three mechanosensory cell types, the pressure ("P") cells [1,2]. But decoding experiments revealed a discrepancy between P cell activity and the behavioral performance [2].

Therefore, we investigated how the mechanosensory touch ("T") cells, respond to touch stimulus properties and influence responses of local bend interneurons. Using a semi-intact preparation, we elicited the local bend response with mechanical stimulation of the skin. Simultaneously, we performed intracellular double recordings from T cells and interneurons 157 or 159, which are involved in the local bend network [3]. We found that T cells respond with characteristic bursts to the onset of touch stimulation. The first T cell spikes are generated with an extremely high temporal precision and short response latency for a broad range of touch stimulus intensities. Local bend interneurons 157 and 159 get input from T cells and produce characteristic EPSPs with short response latencies in response to tactile stimulation. In particular EPSPs of cell 159 follow T cell bursts.

For quantitative data analysis, we used two maximum likelihood approaches of stimulus estimation: The classification approach assigned each response trial to the most probable of all possible stimuli. It provides a measure, how well a certain response feature (e.g. spike count, latency, EPSP amplitude) encodes a specific stimulus property (location, intensity, duration). The pairwise discrimination approach compares responses to two stimuli and quantifies minimal stimulus differences, which could be detected based on the neuronal response features. These methods revealed that the relative latency of two T cells leads to the best estimation of stimulus locations (for tested intensities up to 50 mN). For interneurons, graded response features (e.g. EPSP integral and amplitude) allow good stimulus discrimination performance, even for small distances of touch locations.

Additionally, we started building a computational model of the local bend network. We tried to fit the parameters of the Izhikevich model [4] to intracellularly recorded T cell responses. However, the depolarized resting potential (compared to cortical neurons) of this invertebrate cell required modification of the model. Moreover, we were not able to find a parameter range reproducing the characteristic T-cell bursts in response to stimulus onset. None of the tested parameter sets yielded stimulus-dependent response latencies, which are essential for stimulus encoding. Hence, the Izhikevich model is not well suited for modeling the response properties of an invertebrate sensor cell, which are relevant for the encoding of sensory stimuli.
